# Persistent Liver Manifestations in Allopurinol-Induced Sweet’s Syndrome: An Uncommon Case Report

**DOI:** 10.3390/jcm14207186

**Published:** 2025-10-12

**Authors:** Amalia Papanikolopoulou, Sofia M. Siasiakou, Kosmas Pantazopoulos, Ioannis P Trontzas, Eleni Fyta, Oraianthi Fiste, Ekaterini Syrigou, Nikolaos Syrigos

**Affiliations:** Third Department of Internal Medicine, Sotiria General Hospital, School of Medicine, National and Kapodistrian University of Athens, 11527 Athens, Greece; sofiasiasiakou@yahoo.com (S.M.S.); kospantaz8@gmail.com (K.P.); john-tron@hotmail.com (I.P.T.); elenifita1@gmail.com (E.F.); ofiste@med.uoa.gr (O.F.); esyrigou@hotmail.com (E.S.); nksyrigos@gmail.com (N.S.)

**Keywords:** Sweet’s syndrome, acute febrile neutrophilic dermatosis, liver manifestations, allopurinol, adverse drug reactions

## Abstract

**Background/Objectives:** Sweet’s syndrome (SS), also known as acute febrile neutrophilic dermatosis, is a rare inflammatory skin disorder that may also present with extracutaneous manifestations. Liver involvement is thought to result from sterile neutrophilic infiltration, mirroring the skin pathology and highlighting the syndrome’s systemic inflammatory nature. Timely recognition, exclusion of infectious or autoimmune etiologies, and prompt corticosteroid therapy are critical for favorable outcomes. **Methods:** Herein, we present the case of a 73-year-old man with hyperuricemia who developed both cutaneous and systemic manifestations of SS seven days after initiating allopurinol treatment. His symptoms included fever, conjunctivitis in the right eye, and painful, non-pruritic erythematous plaques, some with pustules, on the lower limbs, palms, and face. **Results:** Initial laboratory investigations revealed neutrophilic leukocytosis, elevated inflammatory markers, and renal and hepatic dysfunction. Empirical treatment with antibiotics and antivirals failed to improve his condition. The patient discontinued allopurinol and initiated a high-dose corticosteroid regimen, leading to rapid resolution of fever and improvement in skin lesions. Laboratory parameters gradually normalized, except for persistent high liver enzymes. A comprehensive diagnostic workup ruled out infectious, autoimmune, and malignant causes. Imaging studies, including CT, MRI, and MRCP, showed no structural liver abnormalities. Skin biopsy findings were consistent with SS, demonstrating dense neutrophilic infiltrates in the reticular dermis and papillary dermal edema. After his discharge, he was followed up by the Hepatology unit. The patients’ liver enzyme levels normalized within three months with no recurrence or late complications one year later. **Conclusions:** In the context of drug-induced SS, persistent hepatic abnormalities, although rare, may occur in patients without underlying liver disease.

## 1. Introduction

Sweet’s syndrome (SS), also referred to as acute febrile neutrophilic dermatosis, is a rare inflammatory condition that typically presents with a sudden onset of fever, leukocytosis, and painful erythematous skin lesions [1]. These lesions commonly appear on the face, neck, upper extremities, and trunk, and histologically demonstrate dense dermal neutrophilic infiltrates without evidence of infection or vasculitis [2].

Although primarily a dermatological entity, SS can involve multiple organ systems, including the eyes, musculoskeletal system, lungs, kidneys, and liver [3]. The pathogenesis remains incompletely understood, although research for this syndrome is ongoing [4,5]. It is believed to involve dysregulated cytokine activity like interleukin-1 (IL-1), IL-6, and granulocyte-colony stimulating factor (G-CSF), which promotes neutrophil activation and tissue infiltration [4]. Particularly, the cytokine IL-17 is involved in SS, with studies showing its expression in the skin lesions of affected patients [5]. Furthermore, the pathogenesis of SS is linked to neutrophil extracellular traps (NETs), which are DNA structures released by neutrophils that form web-like formations and contribute to the inflammatory response observed in SS [5].

Sweet’s syndrome is categorized into three clinical subtypes: classic (idiopathic), malignancy-associated, and drug-induced Sweet’s syndrome (DISS) [3]. The drug-induced form has been associated with several agents, including G-CSF, followed by antibiotics, antiepileptics, and, less frequently, allopurinol [6,7].

While cutaneous manifestations dominate the clinical picture, extracutaneous involvement can occasionally be severe or persistent, particularly in atypical or drug-induced presentations. Among these, hepatic manifestations are rare and typically present as asymptomatic elevations in liver enzymes, although more significant liver injury has been described in patients with predisposing conditions such as autoimmune liver disease, cirrhosis, or viral hepatitis [3,8,9,10].

Here, we present a rare case of allopurinol-induced SS with persistent hepatic laboratory abnormalities in a patient without preexisting liver disease. This case highlights the importance of considering DISS in the differential diagnosis of febrile skin eruptions with multisystem involvement and underscores the need for thorough evaluation and prompt treatment to prevent long-term complications.

## 2. Case Presentation

### 2.1. Case Description

A 73-year-old male presented to the emergency department with an approximate 7-day history of fever up to 39 °C, was hemodynamically stable, and had no oxygen needs. Upon clinical examination, he exhibited conjunctivitis in the right eye, along with painful skin lesions located on his lower limbs, palms, and face for the last 6 days. He also reported right submandibular pain starting 5 days prior. An ultrasound revealed an enlarged right submandibular gland and lymph node. Following the otorhinolaryngology consultation, empirical treatment with clindamycin three times daily was initiated.

His medical history included recent angioplasty with stenting (3 months prior), atrial fibrillation, arterial hypertension, hyperuricemia, and prostatectomy 2 months ago. He had no previous history of drug allergies or reactions, and rejected the use of complementary drugs or alternative medicines. The patient’s chronic medication list included clopidogrel 75 mg daily, apixaban 5 mg twice daily, olmesartan/hydrochlorothiazide 20/12.5 mg daily, rosuvastatin 5 mg daily, flecainide 100 mg ½ tb daily, and metoprolol 100 mg ¼ tb twice daily. Due to elevated serum uric acid (7.2 mg/dL), he was recently started on allopurinol 300 mg daily.

### 2.2. Diagnostic Assessment

**Figure 1:**
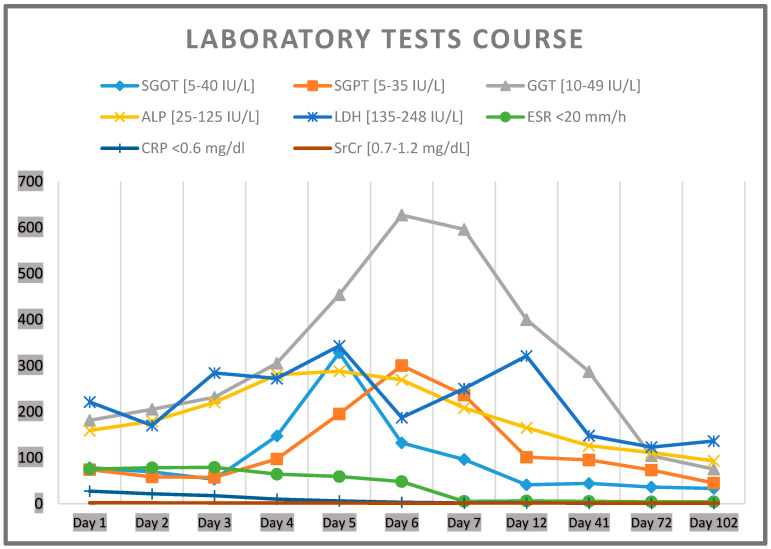
The laboratory test course of the patient. SGOT: serum glutamic–oxaloacetic transaminase; SGPT: serum glutamate–pyruvate transaminase; GGT: gamma-glutamyltransferase; ALP: alkaline phosphatase; LDH: lactate dehydrogenase; ESR: erythrocyte sedimentation rate; CRP: C-reactive protein; SrCr: serum creatinine.

Initial laboratory testing (Day 1) revealed leukocytosis with a leukocyte count of 15.9 (×10^3^/μL), acute kidney injury (SrCr = 1.7 mg/dL, baseline 1.2 mg/dL), and liver enzyme elevations (SGOT = 78 IU/L, SGPT = 74 IU/L, GGT = 181 IU/L, ALP = 159 IU/L) without jaundice. Acute inflammation biomarkers were increased (CRP = 27.25 mg/dL, fibrinogen = 605 mg/dL, ferritin = 725 ng/mL, erythrocyte sedimentation rate (ESR) 75 mm/h), with d-dimers = 6.66 μg/mL and LDH = 221 IU/L, while procalcitonin was negative. The laboratory course is outlined in Figure 1. Due to temporal association, allopurinol was discontinued, and the patient was started on empiric antiviral therapy (acyclovir 400 mg BID), continued clindamycin, and received therapeutic anticoagulation.

**Figure 2:**
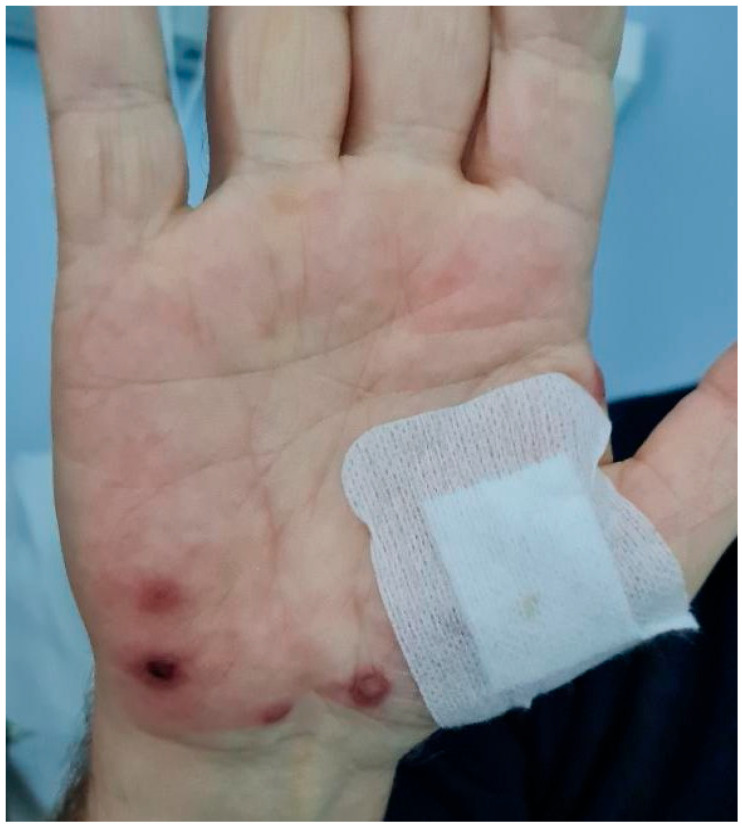
Patient’s skin lesions on right palm presented as red-purple plaques varying in size, often covered by whitish pus-filled lesions (Day 2).

On Day 2, a punch biopsy of the skin was performed on the right palm. The lesions appeared as erythematous to violaceous plaques, some with whitish pustules, and were on both hands with a maximum size of 0.6 cm (Figure 2). Possible differential diagnoses could be 1. Erythema multiforme (infectious), 2. Atypical Stevens–Johnson syndrome (SJS) from allopurinol, and 3. allopurinol-induced Sweet’s syndrome. To exclude infections, blood and urine cultures and pneumococcal urine antigen testing were performed, and all returned negative. Serologies for hepatitis B and C, HIV, EBV, CMV, HSV-1/2, and VZV were negative, as were additional tests for Mycoplasma, Toxoplasma, syphilis, Coxiella, and Coxsackie. Evaluation for autoimmune and liver pathology showed normal serum protein electrophoresis, IgG levels, and 24 h urinary albumin. Screening for autoimmune conditions (ANA, RF, anti-SSA, anti-SSB, c-ANCA, p-ANCA, anti-Scl-70, anti-CCP) was also negative. CT imaging of the chest revealed pleural effusion, while CT of the neck and abdomen was unremarkable, excluding various malignancies.

**Figure 3:**
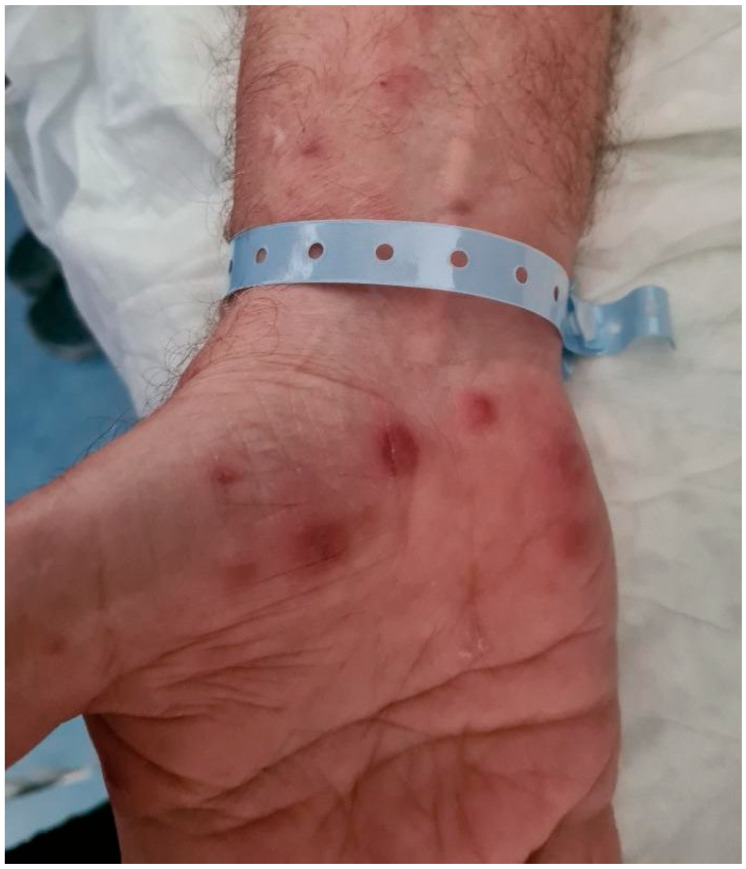
Patient’s skin lesions on right palm (Day 5) after receiving a 3-day course with a high-dose corticosteroid regimen.

On Day 3, based on clinical suspicion for DISS, a high-dose corticosteroid regimen (prednisolone 2 mg/kg) was initiated for three days intravenously. Patient became afebrile, while clindamycin and acyclovir were discontinued. On Day 6, with continued clinical improvement, corticosteroids were tapered over a total 7-day course. The skin lesions demonstrated signs of improvement (Figure 3).

According to the laboratory results, there was a gradual reduction in the inflammation markers and improvement in renal function from day 4. However, the liver laboratory tests indicated daily deterioration after day 5 (Figure 1), prompting the need for complementary abdominal MRI and MRCP, which revealed no pathological findings. Rosuvastatin was temporarily withheld. The result of the skin biopsy was consistent with acute febrile neutrophilic dermatosis (Sweet’s syndrome), with intense neutrophilic dermal infiltrates in reticular dermis and papillary edema. On Day 7, the patient was discharged with instructions for close hepatology follow-up and permanent discontinuation of allopurinol. Liver enzymes normalized approximately 3 months post-discharge, in the absence of any underlying liver disease.

## 3. Discussion

The pathophysiology of SS remains incompletely understood but encompasses complex inflammatory and immune mechanisms. Sweet’s syndrome arises from an aberrant inflammatory response characterized by cytokine-driven neutrophil proliferation, activation, and infiltration into the skin, influenced by various underlying triggers such as infections, malignancies, drugs, and possibly genetic factors [4].

Drug-induced Sweet’s syndrome (DISS) is most commonly triggered by certain medications. The most frequently implicated drug is G-CSF due to its cytokine effect. Other drugs, including trimethoprim-sulfamethoxazole, azathioprine, epidermal growth factor receptor (EGFR) inhibitors, and a variety of chemotherapeutic agents, have also been implicated [6,11]. The typical time frame from drug administration to the appearance of skin lesions was determined to be approximately 5–7 days in most cases [1,5]. In our case, using the Naranjo adverse drug reaction probability scale, a probable relationship was found between the patient’s development of Sweet’s syndrome and allopurinol therapy [12]. To date, only one other case of allopurinol-induced SS has been reported in the literature [7].

In our patient, the presentation aligned with diagnostic criteria for SS, with two major criteria, which are 1. sudden onset of tender plaques or nodules, and 2. skin tissue histology with neutrophilic infiltrate in the dermis without vasculitis; and three minor criteria, which are 1. fever > 38 °C, 2. increased inflammatory markers (CRP/ESR) and white cell count with neutrophil predominance, and 3. positive response to corticosteroids) [3,6]. Additionally, extracutaneous manifestations included right eye conjunctivitis, submandibular gland inflammation, pleural effusion, renal dysfunction, and persistent liver enzyme elevation [1,3].

Allopurinol is a well-established cause of clinically apparent liver injury, mostly with a mixed pattern of liver enzyme elevations and mild and transient serum aminotransferase elevation without jaundice [13,14]. It has been linked to distinctive phenotypes of hepatic adverse reaction: idiosyncratic drug-induced liver injury, drug reaction with eosinophilia and systemic symptoms (DRESS) syndrome, Stevens–Johnson syndrome, toxic epidermal necrolysis, and granulomatous hepatitis [14,15]. The combination of skin biopsy findings, clinical response to corticosteroids, and normalization of liver enzyme tests within 3 months without recurrence 1 year later supported the diagnosis of SS, in the absence of pre-existing chronic liver disease.

The liver involvement in SS is believed to arise from sterile neutrophilic infiltration and inflammation, similar to the skin lesions, reflecting the systemic inflammatory nature of the syndrome [1]. Interestingly, only a few cases have been reported in the literature with SS and liver abnormalities, mostly in patients with underlying liver diseases like chronic hepatitis [8,10,16], liver cirrhosis [8,17,18], or cholangiocarcinoma [19], implying that liver pathology may coexist with or potentially trigger SS. Also, SS can be linked to autoimmune conditions that impact the liver, such as autoimmune hepatitis, where abnormalities in liver enzymes improve with corticosteroid therapy, suggesting an inflammatory process associated with SS [9,20]. The underlying mechanism of liver inflammation could present a common cytokine profile with skin lesions in SS (for instance, IL-1, IL-6, G-CSF) or a distinct one (IL-8, GM-CSF, and IFN-γ) in liver involvement, both leading to neutrophil activation and infiltration [1,3].

In our patient, after extensive exclusion of infections, as well as autoimmune, malignant, and structural hepatic causes, we concluded that the persistent hepatic enzyme abnormalities were most likely due to DISS related to allopurinol. This highlights the importance of maintaining a broad differential and recognizing that drug-induced SS can involve internal organs even in the absence of pre-existing pathology.

## 4. Limitations of the Study

This is a case report showing the occurrence of SS and the administration of allopurinol, along with persistent liver involvement as an extracutaneous manifestation of the syndrome and not the drug itself. There are no reports in the literature with this pharmacovigilance signal in patients without underlying liver disease. Healthcare professionals should be aware of this possibility and be encouraged to investigate and report any relevant cases they observe. Findings of such case reports cannot be generalized. Larger case series or systematic studies are needed to determine the frequency and mechanisms of liver involvement in drug-induced SS.

## 5. Conclusions

Liver involvement in SS may arise merely from sterile neutrophilic infiltration and inflammation, mirroring the dermatologic lesions and underlining the syndrome’s systemic inflammatory profile. Prompt recognition and initiation of corticosteroid therapy are essential for symptom resolution and to avoid complications. Importantly, hepatic manifestations may persist beyond the resolution of cutaneous symptoms, even in the absence of underlying liver disease, particularly in drug-induced cases. This underscores the need for vigilant monitoring and a multidisciplinary approach in managing atypical or systemic SS presentations.

## Data Availability

Data are contained within the article.

## Abbreviations

The following abbreviations are used in this manuscript:

SS	Sweet’s syndrome
IL-1	Interleukin-1
G-CSF	Granulocyte-colony stimulating factor
DISS	Drug-induced Sweet’s syndrome
SGOT	Serum glutamic–oxaloacetic transaminase
SGPT	Serum glutamate–pyruvate transaminase
GGT	Gamma-glutamyltransferase
ALP	Alkaline phosphatase
LDH	Lactate dehydrogenase
CRP	C-reactive protein
SrCr	Serum creatinine
ESR	Erythrocyte sedimentation rate
BID	Twice daily
SJS	Stevens–Johnson syndrome
EGFR	Epidermal growth factor receptor inhibitors
DRESS	Drug reaction with eosinophilia and systemic symptoms
G-MCSF	Granulocyte-macrophage colony-stimulating factor
INF-γ	Interferon-γ
